# Beech Wood Pyrolysis in Polyethylene Melt as a Means of Enhancing Levoglucosan and Methoxyphenol Production

**DOI:** 10.1038/s41598-018-37146-w

**Published:** 2019-02-13

**Authors:** Shogo Kumagai, Kohei Fujita, Yusuke Takahashi, Yumi Nakai, Tomohito Kameda, Yuko Saito, Toshiaki Yoshioka

**Affiliations:** 1 Graduate School of Environmental Studies, Tohoku University, 6-6-07 Aoba, Aramaki-aza, Aoba-ku, Sendai, Miyagi 980-8579 Japan; 2 JEOL RESONANCE, Inc., 3-1-2 Musashino, Akishima, Tokyo 196-8558 Japan

## Abstract

Recycling wood/plastic composites in municipal and industrial wastes currently represents a challenge which needs to be overcome. In this work, we considered the concept of independent pyrolysis of wood and plastic in wood/plastic mixtures for enabling a versatile catalytic process design which is capable of producing recoverable final products from both components. In order to reveal the influence of plastic on wood pyrolysis, the pyrolysis of beech wood (BW, wood material) in a polyethylene (PE) melt (polyolefin material) was performed at 350 °C. The combined use of thermogravimetric analysis, product recovery studies, *in situ* radical characterisations, and microscopic analysis revealed the influence of the PE melt on the BW pyrolysis. More specifically, a physical prevention of the intermolecular condensation and hydrogen abstraction from PE pyrolysates in the liquid/solid phase was observed. These interactions enhanced the production of levoglucosan and methoxyphenols by factors of 1.7 and 1.4, respectively, during the BW pyrolysis in the PE melt. Based on these results, we concluded that the observed synergistic effects could potentially control the yield and quality of useful products, as well as the utilisation of mixed wood/plastic wastes, which cannot be effectively recycled otherwise.

## Introduction

Wood/plastic composites have recently attracted increased amount of attention because they feature the combined advantages of both constituents, e.g., corrosion resistance, high stiffness, and excellent injection-molding processability^1^. Additionally, the broad availability and renewability of wood as a resource is another important benefit. Unsurprisingly, the demand for these composites, which are often used for outdoor decking, automotive parts, and furniture, is steadily increasing^1^, as exemplified by a novel and lightweight cellulose nanofiber–reinforced resin which exhibits excellent mechanical properties and is thus suitable for automotive or aerospace applications^2^. Waste management processes commonly generate wood/plastic mixtures from municipal solid and construction wastes. Although these mixtures are promising carbon resources for the production of fossil fuel alternatives, their complete separation into biomass and plastic by physical means is both technologically and economically difficult, and most of these mixtures are therefore disposed of in landfills or by incineration, the latter of which could be with or without energy recovery.

Pyrolysis achieves chemical bond cleavage in polymeric materials by the action of heat alone, thus exhibiting a distinct advantage over selective solvolysis-induced depolymerisation, as it is applicable to material mixtures which cannot be easily separated. In view of this fact, the co-pyrolysis of wood/plastic mixtures has been extensively investigated to reveal synergistic interactions between biomass and plastics^3–21^. For example, Marin *et al*.^4,6^ investigated the co-pyrolysis of beech wood (BW)/polypropylene (PP) and BW/polyethylene (PE) mixtures, achieving enhanced oil production from PP due to its accelerated pyrolysis by the BW radicals generated at low temperatures. Moreover, Brebu *et al*.^10^ reported that tar production from cedar wood is enhanced by PE, PP, and polystyrene (PS) addition, because these plastics donate hydrogen to the cedar wood pyrolysates during co-pyrolysis. Finally, it has also been reported that 1,6-anhydro-β-D-glucopyranose (levoglucosan, LG) and methoxyphenol radicals are stabilised by hydrogen abstraction from hydrogen-rich PE pyrolysates in the vapour phase^19^.

Co-pyrolysis is often followed by catalytic conversion to obtain value-added compounds^22^. Previously, synergistic effects have been observed for the catalytic conversion of wood/plastic pyrolysates. For example, cellulose/PP mixtures afforded increased amounts of olefins and alcohols in the presence of MCM-41 mesoporous silica^23^, with increased aromatic hydrocarbon production observed for cellulose/PE and cellulose/PP in the presence of zeolites^24–26^ and for lignin/low-density PE and lignin/PP mixtures in the presence of HZSM-5 zeolite^27,28^. Moreover, similar effects were also detected for wood/plastic mixtures in the presence of various zeolites^29,30^, and plastic-addition-enhanced production of H_2_-rich syngas was observed for the steam gasification of wood biomass using supported noble metal catalysts^31–33^.

Thus, the synergistic effects in wood/plastic co-pyrolysis and the catalytic conversion of the corresponding pyrolysates have been widely researched, with the former helping to control the reaction and product selectivity and thus providing the desired products for subsequent catalytic processes, and the latter being important for maximizing the yield and quality of the final products. Nevertheless, the simultaneous generation of wood- and plastic-derived complex pyrolysates is inevitable in both cases, complicating the design of catalytic conversion processes and limiting the versatility of the final products.

**Figure 1:**
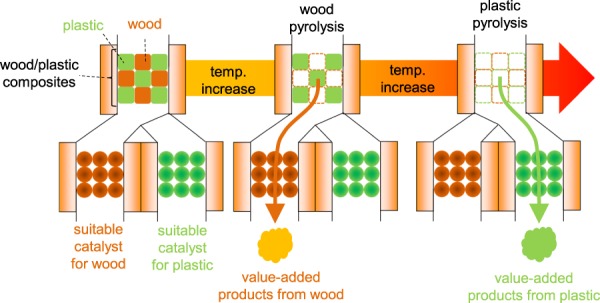
Concept of independent pyrolysis of wood and plastic in wood/plastic mixtures.

In the present study, we considered the concept of the independent pyrolysis of wood and plastic in wood/plastic mixtures (Fig. 1). In general, wood is pyrolysed at lower temperatures than common polyolefins such as PE and PP, which melt at the temperature required for wood pyrolysis. Therefore, the independent pyrolysis of wood and plastic could theoretically be realised by temperature control, allowing for the selective generation of wood-derived oxygen-containing compounds and olefinic polymer-derived aliphatic compounds in different temperature ranges. Therefore, this approach expands the versatility of catalytic process design and broadens the scope of the final products, enabling the use of different catalysts and conditions for wood and plastic pyrolysis^34–37^.

**Figure 2:**
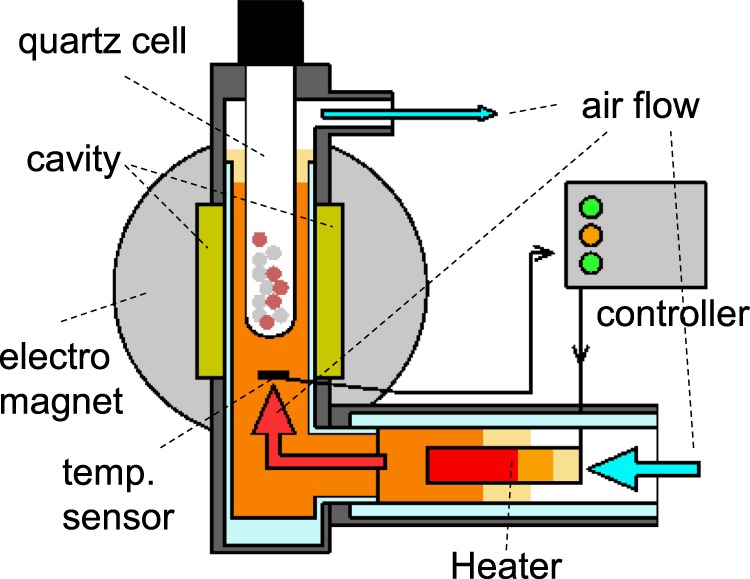
Side view of the ESR spectrometer featuring a novel heating unit.

In order to investigate the feasibility of the aforementioned approach, we studied wood pyrolysis in a plastic melt, with BW and PE used as representatives of wood and plastic, respectively. To the best of our knowledge, the interactions between wood and plastic melt have never been investigated. Therefore, in the present study, we evaluated the synergistic effects of co-pyrolysis by pyrolysing BW and PE both individually and as components of various BW/PE mixtures, using a tube reactor for product identification and quantification. Additionally, we employed a thermogravimetric analyser equipped with a charge-coupled device (CCD) camera to simultaneously observe the weight loss behaviour and sample changes. Furthermore, an electron spin resonance (ESR) spectrometer featuring a novel heating unit (Fig. 2) was utilised to characterise the *in situ* radical behaviour.

## Results and Discussion

### TGA measurements of the BW/PE mixtures

**Figure 3:**
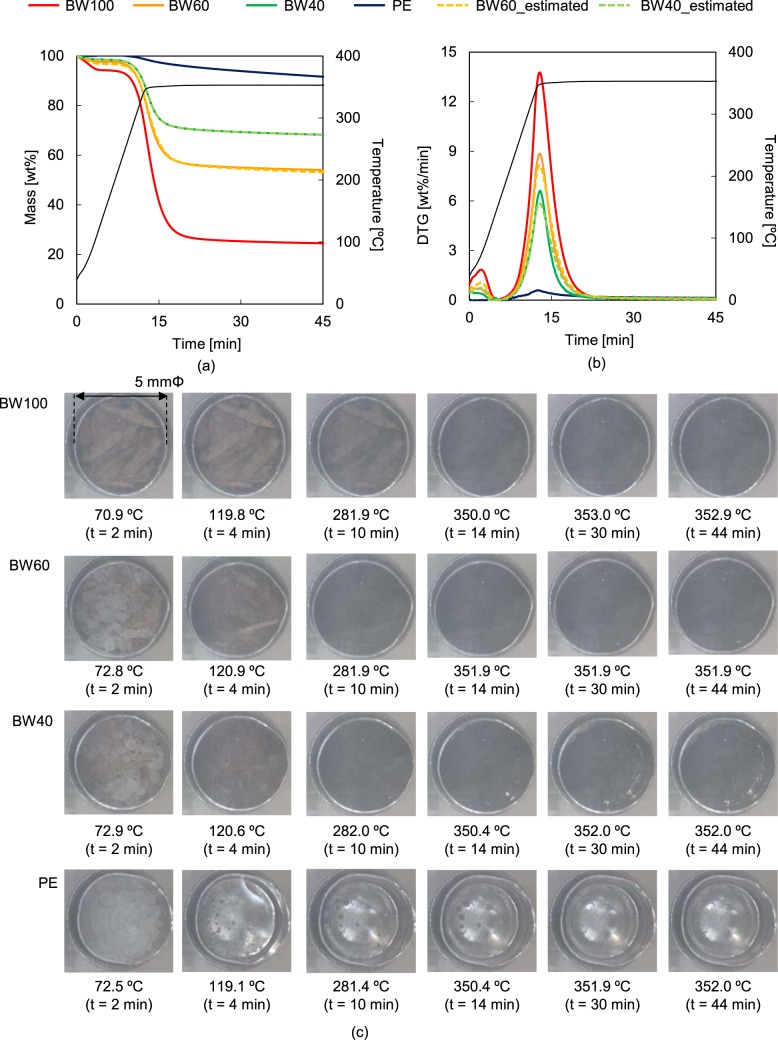
Experimental and estimated (**a**) TGA and (**b**) DTG curves, and (**c**) pictures of BW, PE, and their mixtures recorded at 350 °C.

Figure 3(a,b) display the experimental and estimated TGA and derivative TG (DTG) curves of the BW/PE mixtures, respectively, while Fig. 3(c) shows pictures taken during the pyrolysis. During the measurements, the sample temperature reached 350 °C within 15 min and that temperature was maintained for 30 min. The weight loss of BW was almost complete within 15 min after reaching 350 °C, resulting in 24.5 wt% char after 45 min. The BW pictures clearly show the significant colour change of the sample from brown to black through a temperature range of 300 °C due to carbonisation. In contrast, 91.6 wt% of the PE remained after 45 min of the experiment. PE melted at 108 °C (confirmed by differential thermal analysis (DTA)) and the pictures reveal that the PE melt remained intact at 350 °C. These results show that the present pyrolysis conditions (350 °C, 30 min) were well suited to the almost complete pyrolysis of BW, with the extent of PE decomposition being only 8.4 wt%.

These conditions were then applied to the pyrolysis of the BW60% and BW40% samples. The resulting experimental TG and DTG curves were almost the same to the estimated ones, suggesting the lack of strong influence on the weight loss during co-pyrolysis. Additionally, the pictures revealed that PE melted initially, followed by the BW pyrolysis in the PE melt. As can be noticed from the photos, the colour of the BW in the PE melt at 121 and 282 °C is darker than that of neat BW. As opposed to the previous case, the wet colour of the BW is due to the PE penetration into the BW, not due to carbonisation.

### Pyrolysis of the individual PE and BW components in tube reactor

The identified pyrolysates are categorised in Table 1, which also lists their weight compositions, with detailed breakdowns for each category provided in Table S2. Notably, 89.7 wt% of the PE remained in the sample holder after the pyrolysis run, which was in agreement with the TGA results (Fig. 3(a)). Thus, only 0.4 wt% of gas and 0.4 wt% of oil were identified from PE under the chosen conditions, with the remaining 9.5 wt% mainly attributable to the high molecular weight oil which is not detected by GC and wax. Conversely, BW pyrolysis produced 4.1 wt% gas, 12.7 wt% tar, 10.8 wt% water, and 26.5 wt% char. The BW tar mainly consisted of cellulose-derived LG (**1**), with its total weight fraction equalling 5.6 wt%. Moreover, cellulose and LG pyrolysis also afforded gases such as CO, CO_2_, and hydrocarbons, and tars such as anhydrosugars (**2**), C_2_–C_4_ fragments (**3**), and five-membered ring compounds (**4**)^38–40^. However, some of these compounds were also produced from hemicellulose^41^, whereas lignin pyrolysis afforded diverse substituted methoxyphenols (**5**–**7**) and simple phenols (**8**).

**Table 1:** Weight compositions of the pyrolysates obtained from PE, BW, and BW/PE mixtures at 350 °C.

	(BW + PE)-based weight composition, *F*_W_				BW-based weight composition, *F*_W, std_^a^	
**BW:PE (w:w)**						
	0:100	40:60	60:40	100:0	40:60	60:40
**Weight-based mass balance/wt%**						
Gas^b^	0.4 ± 0.0^f^	2.2 ± 0.1	2.7 ± 0.1	4.1 ± 0.1	−	−
THF soluble^c^	6.7 ± 1.1	26.7 ± 1.3	38.3 ± 1.4	61.4 ± 1.1	−	−
THF insoluble^c^	2.6 ± 1.0	4.6 ± 1.0	7.1 ± 1.1	7.0 ± 1.0	−	−
Melted PE + char^c^	89.7 ± 1.9	65.6 ± 1.6	51.4 ± 1.5	26.5 ± 1.0	−	−
**Total/wt%**	**99**.**4** ± 3.0	**99**.**1** ± 4.0	**99**.**5** ± 4.1	**99**.**0** ± 3.2	−	−
**Mass balance of identified products/wt%**						
*Gas* ^b^	*0*.*4* ± *0*.*0*	*2*.*2* ± *0*.*1*	*2*.*7* ± *0*.*1*	*4*.*1* ± *0*.*1*	*/*	*/*
CO	−	0.4 ± 0.0	0.6 ± 0.0	1.0 ± 0.0	0.9 ± 0.0	1.0
CO_2_	−	1.5 ± 0.0	1.9 ± 0.0	3.0 ± 0.0	3.7 ± 0.0	3.1
CH_4_	−	−	+	+	−	+
C_2_	+	+	+	+	/	/
C_3_	0.1 ± 0.0	+	+	+	/	/
C_4_–	0.2 ± 0.0	0.2 ± 0.0	0.2 ± 0.0	0.1 ± 0.0	/	/
*Tar* ^b^	−	*7*.*1* ± *0*.*5*	*9*.*8* ± *0*.*7*	*12*.*7* ± *1*.*5*	*17*.*8* ± *1*.*3*	*16*.*3* ± *1*.*2*
**1**	−	3.7 ± 0.4	4.8 ± 0.5	5.6 ± 0.6	9.3 ± 1.0	8.0 ± 0.8
**2**	−	0.1 ± 0.0	0.2 ± 0.0	0.4 ± 0.1	0.3 ± 0.0	0.4 ± 0.0
**3**	−	0.2 ± 0.1	0.5 ± 0.1	1.0 ± 0.1	0.5 ± 0.3	0.8 ± 0.2
**4**	−	0.3 ± 0.1	0.5 ± 0.0	0.8 ± 0.1	0.6 ± 0.2	0.8 ± 0.0
**5**	−	0.1 ± 0.0	0.1 ± 0.0	0.1 ± 0.0	0.2 ± 0.0	0.2 ± 0.0
**6**	−	0.2 ± 0.0	0.2 ± 0.0	0.3 ± 0.0	0.5 ± 0.0	0.4 ± 0.0
**7**	−	0.3 ± 0.0	0.6 ± 0.0	0.7 ± 0.1	0.8 ± 0.0	0.9 ± 0.1
**8**	−	+	+	+	+	+
others	−	2.2 ± 0.1	2.9 ± 0.1	3.7 ± 0.1	5.5 ± 0.3	4.8 ± 0.1
*Oil* ^b^	*0*.*4* ± *0*.*1*	*0*.*3* ± *0*.*0*	*0*.*3* ± *0*.*0*	−	*/*	*/*
*Melted PE* ^3^	*89*.*7* ± *1*.*9*	−	−	−	*/*	*/*
*Char* ^c^	−	−	−	*26*.*5* ± *1*.*0*	*/*	*/*
*Melted PE* + *Char*^3^	−	*65*.*6* ± *1*.*6*	*51*.*4* ± *1*.*5*	−	*/*	*/*
*Water* ^d^	−	*5*.*3* ± *0*.*1*	*8*.*7* ± *0*.*1*	*10*.*8* ± *0*.*3*	*/*	*/*
**Identified total** ^e^	**90**.**5 ± 2**.**0**	**80**.**5 ± 2**.**3**	**72**.**9 ± 2**.**4**	**54**.**1 ± 2**.**4**	**/**	**/**

–not detected; +: < 0.05 wt%;/skipped calculation.

^a^Calculation was carried out for compounds derived only from BW.

^b^Determined by GC analysis.

^c^Determined by weight measurement.

^d^Determined by the Karl Fischer titration.

^e^Unidentified products include wax and high−molecular-weight compounds (THF insoluble, undetectable by GC).

^f^Deviation less than 0.05.

### Pyrolysis of the BW/PE mixtures in the tube reactor

**Figure 4:**
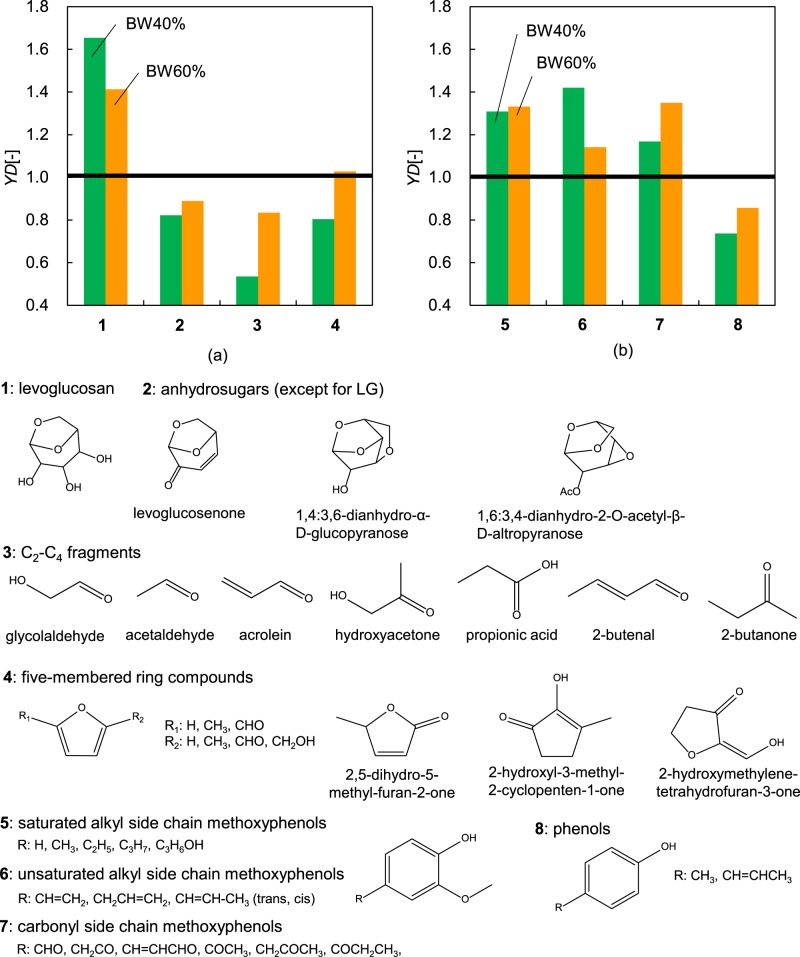
Yield differences of the tar compounds derived from (**a**) (hemi)cellulose and (**b**) lignin.

The BW-based weight fractions (*F*_W,std_) of the obtained products are summarised in Table 1, while the *YD* values of the tars are summarised in Fig. 4. The *F*_W,std_ values were calculated for tar compounds produced only from BW. Interestingly, co-pyrolysis with PE increased the yields of BW tar, which equalled 17.8 and 16.3 wt% for BW40% and BW60%, respectively. Cellulose-derived **1** and lignin-derived **5**–**7** contributed to the above increase in the tar yield, whereas compounds 2–4 and 8 resulted in *YD* < 1. The *YD* of **1** was maximal (1.7) for BW40%, decreasing to 1.4 for BW60%. In contrast, the formation of **2**–**4**, which are the main pyrolysates of **1**, was largely suppressed during the BW pyrolysis in the PE melt, with their *YD* values decreasing with the increasing PE content. Therefore, this behaviour indicates that the decomposition of **1** was suppressed during the co-pyrolysis, whereas that of lignin was enhanced, affording products **5**–**7** with *YD* values of 1.1–1.4. In contrast, the *YD* of **8**, which is a pyrolysate of **5**–**7**, equalled 0.7–0.9, suggesting that the decomposition of these compounds is suppressed during co-pyrolysis.

Notably, the enhanced production of **1** and **5**–**7**, as well as the decreased formation of LG- and methoxyphenol-derived pyrolysates, has been reported for BW/PE mixture pyrolysis at temperatures of up to 650 °C^19^. In this work, the total tar yield was enhanced by the BW pyrolysis in the PE melt, while maintaining the tar composition comparable to that obtained at temperatures of up to 650 °C. In addition, when the aforementioned conditions were employed, the production of gases, mainly CO and CO_2_, was suppressed. Hence, it can be concluded that the BW pyrolysis in the PE melt was beneficial to the formation of both LG and methoxyphenols at lower temperatures while simultaneously avoiding the mixing of PE pyrolysates.

Since the emission of PE pyrolysates into the vapour phase under the conditions used in the present study was insignificant, it was concluded that the observed synergistic effect, which enhanced the formation of **1** and **5**–**7**, originated in the liquid/solid phase.

### Pyrolysis in the ESR apparatus

**Figure 5:**
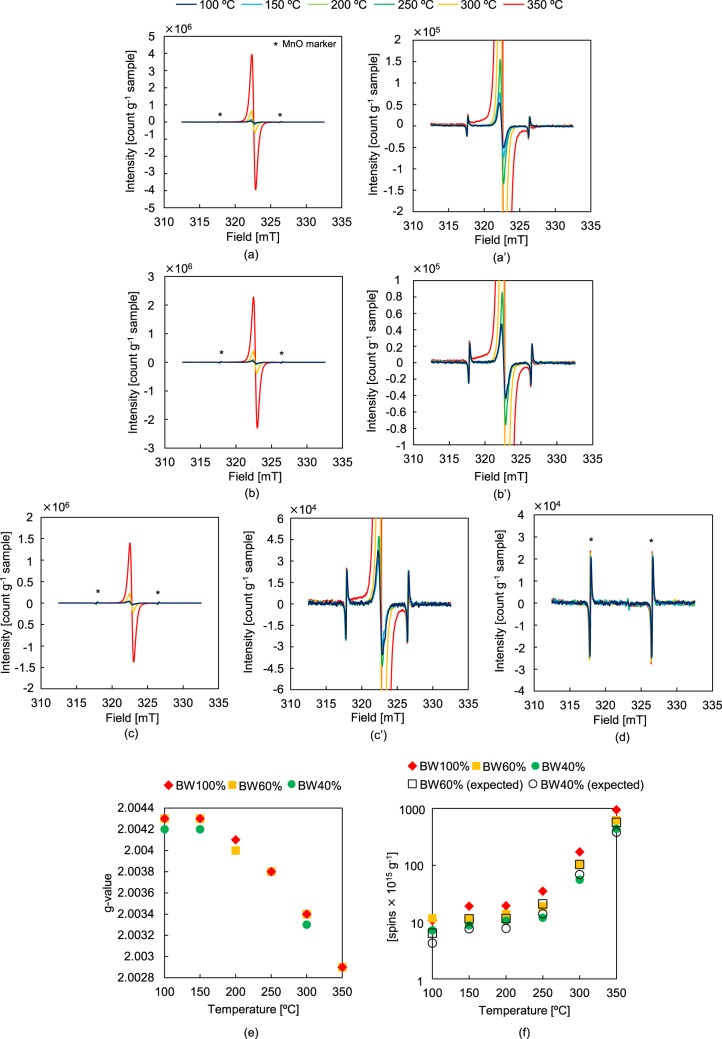
ESR spectra of liquid/solid phases recorded during the pyrolysis of (**a**) BW100%, (a′) BW100% (magnified), (**b**) BW60%, (b′) BW60% (magnified), (**c**) BW40%, (c′) BW40% (magnified), and (**d**) BW0%; (**e**) *g* values obtained under different conditions; (**f**) experimental or theoretical spin concentrations obtained under different conditions.

In order to investigate the behaviour of radicals in the liquid/solid phases during BW/PE pyrolysis, individual PE and BW components and their mixtures were pyrolysed in an ESR spectrometer equipped with a high-temperature heating unit. The obtained results are summarised in Fig. 5. The acquired data indicates that radicals for which *g* = 2.0043 were already formed at 100 °C (Fig. 5(c)), corresponding to oxygen-containing species such as BW lignin-derived phenoxy and semiquinone radicals^42,43^. This conclusion was supported by the fact that no peaks were detected during the *in situ* ESR analysis of cellulose up to 200 °C (Figure S4(a)) and that the bond cleavage of *O*-acetyl-(4-*O*-methylglucurono)xylan, which is the principal hemicellulose framework component of BW, proceeds via an ionic mechanism^44,45^. When the measurement temperature was increased from 100 to 150 °C, the spin concentration almost doubled (from 1.1 × 10^16^ to 1.9 × 10^16^ spin g^−1^ (Fig. 5(f))), whereas the *g* value remained constant. Above 250 °C, the spin concentration increased with the temperature, since the homolytic cleavage of chemical bonds in BW lignin is reported to start at 250 °C^41^, thereby causing the subsequent radical chain reactions to progress. The slight decrease in *g* to 2.0041 at 200 °C was ascribed to the influence of mono- and dimethoxybenzenes generated by lignin pyrolysis (*g* = 2.0035–2.0039)^43^, with the drastic decrease to 2.0034 at 300 °C being attributed mainly to the enhanced homolytic cleavage of lignin *β*-aryl-ether bonds. Even though the degradation of *O*-acetyl-(4-*O*-methylglucurono) xylan is significant at this temperature^41^, bond cleavage mainly proceeds via an ionic mechanism^44,45^. Moreover, cellulose decomposition at this temperature is insignificant^46,47^, mainly occurring by dehydration (i.e., via an ionic pathway)^48^ and thus having no influence on the ESR results. At 350 °C, *g* was equal to 2.0029, corresponding to carbon-centred radicals such as one- to five-ring aromatic hydrocarbon and graphitic carbon radicals^49,50^ which originated mainly from the char. Pastorova *et al*.^51^ reported that cellulose-derived char produced at 350 °C mainly exhibits an aromatic structure, implying that similar behaviour can be expected for the hemicellulose framework. Therefore, the significant increase in the spin concentration (to 9.5 × 10^17^ spin g^−1^) at 350 °C was ascribed to the contribution of char originating not only from the lignin but also from the cellulose and hemicellulose. Thus, the novel heating unit allowed us to evaluate the *in situ* behaviour of pyrolytically produced radicals at high temperature.

Notably, no detectable ESR spectrum was observed for PE at any temperature. This suggests that the radicals produced from PE were short-lived alkyl radicals^52^, which were hard to detect under the given ESR conditions^53^. It is also expected that the quantity of radicals generated would not be significant because of the slow PE pyrolysis rate at 350 °C observed by the TG analysis and tube-reactor experiment. In fact, we confirmed the carbon-centred radicals (*g* = 2.0031) during the PE pyrolysis at 400 °C (Fig. S4(b) in the Supporting Information (SI)).

The measured spin concentrations for BW60% and BW40% were comparable to the expected values calculated from pure BW since no PE radicals were detected. By the same reasoning, the *g* values of the mixed samples were comparable to those determined for pure BW and pure PE. Thus, these results suggest that the formation of BW radicals (amount and species) was not influenced by the PE melt.

### Microscopic investigation of pristine and pyrolysed BW

**Figure 6:**
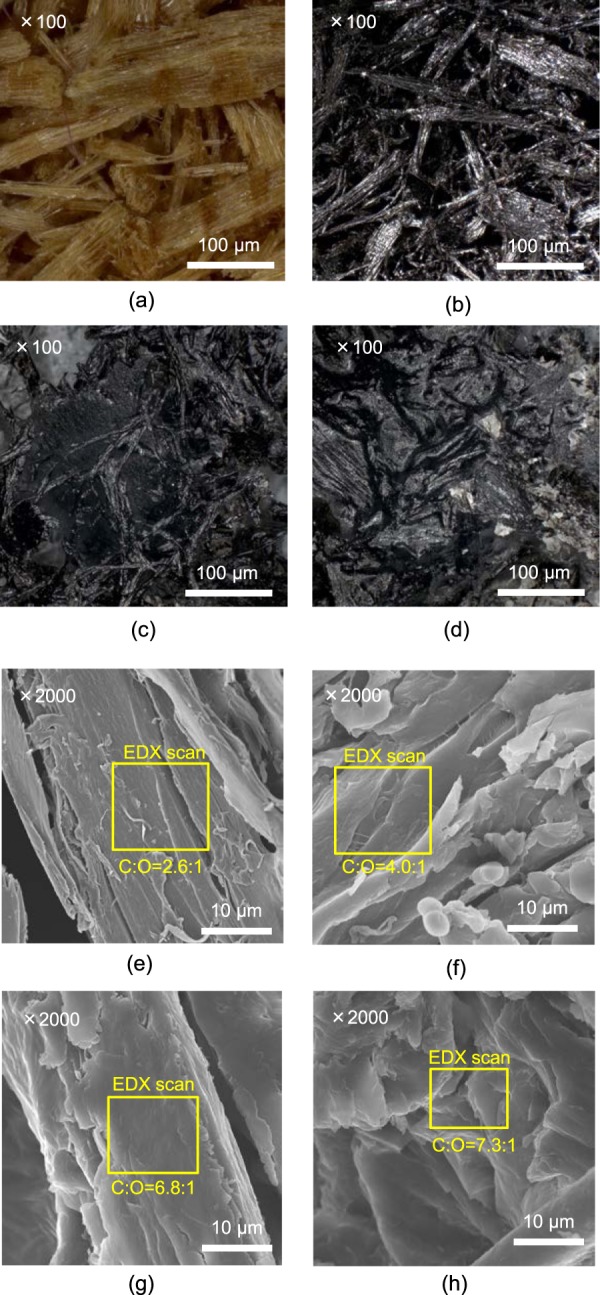
Microscopic images of (**a**) the original BW, (**b**) BW char obtained from BW100%, and BW char coated by PE-melt obtained from (**c**) BW60% and (**d**) BW40%. SEM images of (**e**) original BW, (**f**) BW char obtained from BW100%, and BW char coated by PE-melt obtained from (**g**) BW60% and (**h**) BW40%.

Pristine and pyrolysed BW were analysed by optical microscopy and SEM (Fig. 6). In the presence of PE, the BW primary char was apparently coated by a polyolefin (Fig. 6(c,d)). As can be seen, there was no clear difference between the SEM images of the BW char surface obtained in the presence and absence of PE (Fig. 6(e–h)). In contrast, the C/O atomic ratios of the char surface obtained by EDX revealed an increase in the value of the original sample after pyrolysis (from 2.6 to 4.0; Fig. 6(e)) due to the char formation. Moreover, the C/O ratio increased with the PE content, equalling 6.8 and 7.3 for BW60% (Fig. 6(g)) and BW40% (Fig. 6(h)), respectively. These results support the hypothesis of a PE coating around the BW char (Fig. 6(c,d)). However, it should be noted that the abovementioned values are not completely accurate due to the irregular sample surface and limited penetration depth of the electron beam (around 4 μm at 20 kV), thereby implying the significance of the increase in the C/O ratio upon increasing the PE amount. Based on the aforementioned results, it is clear that the BW particles were dispersed and pyrolysed in the PE melt under the described pyrolysis conditions.

### Suggested mechanism

In summary, the pyrolysis of BW in the PE melt (i) enhanced the production of LG (**1**) and inhibited the production of decomposition products (**2**–**4**) of **1**, and (ii) enhanced the production of methoxyphenols (**5**–**7**) and inhibited that of phenols (**8**). These synergistic effects obtained by mixing could be respectively explained by (I) the physical blocking of the hydrogen bond formation by the PE melt and (II) the hydrogen abstraction from the PE pyrolysates.

#### Physical blocking of the hydrogen bond formation

**Figure 7:**
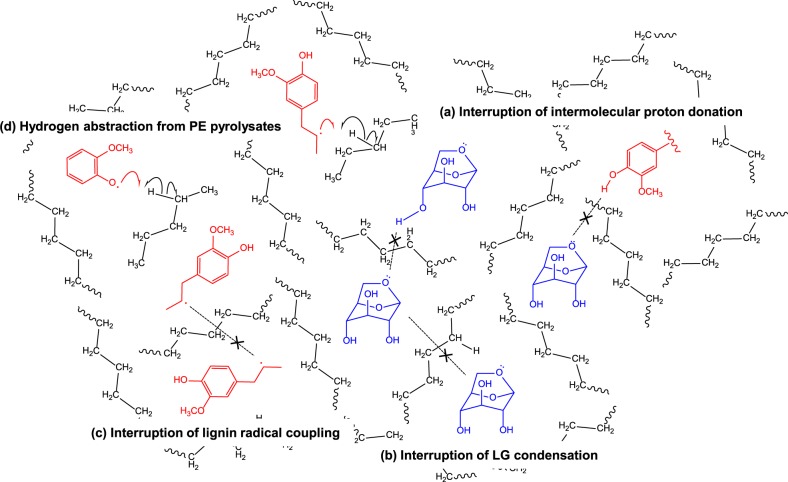
Suggested interactions during the BW pyrolysis in the PE melt: (**a**) interruption of intermolecular proton donation, (**b**) interruption of condensation of **1**, (**c**) interruption of coupling between lignin radicals, and (**d**) hydrogen abstraction from hydrogen-rich PE pyrolysates.

The PE melt mainly influenced the BW pyrolysis by facilitating BW particle dispersion. Specifically, early-stage pyrolysates such as **1** were dispersed in the PE melt (Fig. 7), with liquid-phase 1 being further converted to **2**–**4** via ring-opening reactions^39,40^. In line with this explanation, Kawamoto *et al*.^54^ reported on the acid-catalysed ring opening of 1 during its pyrolysis in the molten state by proton donation to the C_1_-oxygen via intermolecular hydrogen bond formation. Importantly, the PE melt could physically interrupt intermolecular proton donation from other molecules (Fig. 7(a)) and thus block the acid-catalysed ring opening of **1**, as confirmed by the decreased production of **2**–**4** (pyrolysates of 1) in the liquid and solid phases in the presence of PE (Fig. 4). In addition, the dispersion of **1** in the PE melt physically prevented intermolecular condensation of **1** (Fig. 6(b)), thereby enhancing the recovery of **1**.

#### Hydrogen abstraction from the PE pyrolysates

Lignin-derived radicals generally couple to form dimers or condensation products, which are further cracked to form **8** via alkyl side chain elimination. The radicals derived from **5–7** are dispersed in the PE melt, which physically interrupts the radical coupling (Fig. 7(c)). Furthermore, these radicals could abstract hydrogen from the hydrogen-rich PE pyrolysates (Fig. 7(d)), resulting in the enhanced generation of **5**–**7** and suppression of the formation of **8**.

Thus, the present work reveals the influence of the PE melt on BW pyrolysis: specifically, the presence of the melt physically prevents intermolecular condensation, while the BW pyrolysates abstract hydrogen from the hydrogen-rich PE pyrolysates in the liquid/solid phase. Moreover, this study contributes to the practical realisation of the independent pyrolysis of wood and plastics in a wood/plastic mixture, thereby paving the way to achieving versatile catalytic process design and increasing the yield of the desired pyrolysates.

## Methods

### Materials

Commercial BW and PE (Sigma-Aldrich, Tokyo, Japan) were ground and sieved to a particle size of <250 μm. The properties of BW^19^ are summarised in Table S1 (Supporting Information, SI). Moisture-, extractives-, and ash-free BW^55,56^ was used in all experiments to avoid the influence of these components during pyrolysis. The characterisation and sample preparation procedures are summarised in the SI.

### TGA measurements

The weight-loss behaviours of BW, PE, and their mixtures, as well as the simultaneous CCD camera observations of the sample changes were obtained using a TG analyzer, STA7200RV (Hitachi High-Tech Science Corporation, Tokyo, Japan). The CCD camera was located at the upper outside part of the quartz furnace in order to take sample pictures from the top through the quartz furnace. Each sample (10 mg) was loaded in a Pt pan, while a sapphire plate (10 mg) was used as a reference. After placing the pan into the instrument, the device was purged with N_2_ at a flow rate of 200 mL/min for 10 min. Then, the temperature was raised from ambient to 350 °C at a heating rate of 25 °C/min. The samples were kept at that temperature for 30 min under N_2_ flow. The temperature and experimental time were was set to these values as they allowed an almost complete BW pyrolysis with minimal PE pyrolysis, as thermogravimetrically confirmed (Figs 3(a) and S2). The sample pictures were captured by every 2 min.

The experimental TGA curves (*Y*_TG_exp, *t*_ [wt%]) and DTG curves (*Y*_DTG_exp, *t*_ [wt%/min]) *vs*. time (*t* [min]) were compared using estimated plots (*Y*_TG_cal, *t*_ *vs*. *t* and *Y*_DTG_cal, *t*_ *vs*. *t*) determined via Eq. (1):

1 $${Y}_{\mathrm{TG\; or\; DTG}\_\mathrm{cal},t}={Y}_{{\rm{TGorDTG}}\_\exp \_\mathrm{BW},t}\times {R}_{{\rm{BW}}}/100+{Y}_{{\rm{TGorDTG}}\_\exp \_\mathrm{PE},t}\times {R}_{{\rm{PE}}}/100$$

where *Y*_TG or DTG_exp_BW or PE, *t*_ are the TGA or DTG values of neat BW or PE at time *t*, while *R*_BW or PE_ [wt%] are their respective mixing ratios.

### Pyrolysis in the tube reactor

Pyrolysis was carried out in a horizontal quartz tube reactor heated by an electric furnace (Fig. S2). Samples (0.2 g) with BW:PE (w:w) ratios of 100:0, 60:40, 40:60, and 0:100 were prepared and loaded into a ceramic sample holder located outside the heating zone of the quartz tube reactor. The reactor was purged with helium at a flow rate of 100 mL/min, which was controlled by a digital mass flow controller (FCST1000L, Fujikin, Osaka, Japan), and the temperature was set to 350 °C. Subsequently, the sample holder was pushed into the centre of the heating zone, where the samples were pyrolysed for 30 min. The sample temperature reached 350 °C for ~100 s. The pyrolysis products were condensed in a liquid-nitrogen-cooled trap or collected in an aluminium bag.

After 30 min, heating was stopped, and the tube reactor was cooled down to ambient temperature under continuous helium flow. The cooling trap was defrosted in a water bath, with He gas being passed through the trap for another 5 min to allow the transfer of any condensed gases such as CO_2_ and CH_4_ into the aluminium collection bag. The inside surfaces of the trap and reactor walls were washed with super-dry THF (20 mL) to remove the condensed products. Wax, i.e., a THF-insoluble sticky solid derived from PE, was not quantified since it was formed in minute amounts. The mixture of BW char and PE remaining in the sample holder was weighed, whereas the secondary char (deposited on the reactor wall) was not quantified as it was insoluble in THF. The products, which were identified and quantified using gas chromatography (GC), could be categorised into: a gas which was collected in an aluminium bag, tar which was a BW-derived tetrahydrofuran (THF)-soluble liquid, and oil which was a PE-derived THF-soluble liquid. The detailed procedure and analytical conditions for the GC analysis are summarised in the SI. The water yield was determined by the Karl Fischer titration method. The char and PE melt were analysed by optical microscopy (VHX-2000, Keyence, Japan) and scanning electron microscopy coupled with energy-dispersive X-ray spectroscopy (SEM-EDX; S-4800, Hitachi, Japan). Reliable mass balances were obtained after all experiments and are summarised in the upper part of Table 1.

The weight fractions of the decomposition products (*F*_W_ [wt%]) were calculated as

2 $${F}_{{\rm{W}}}[\mathrm{wt} \% ]=\frac{{W}_{\exp }}{{W}_{{\rm{sample}}}}\times 100$$

where *W*_sample_ [g] and *W*_exp_ [g] are the input sample and product weights, respectively.

The synergistic effect of co-pyrolysis on the BW pyrolysate production was evaluated by determining the yield difference (*YD* [-]) as

3 $$YD[-]=\frac{{F}_{W,{\rm{std}}}}{{F}_{W,\mathrm{BW100} \% }}$$

where *F*_W, BW100%_ [wt%] is the weight fraction of a given product obtained by pure BW pyrolysis and *F*_W,std_ [wt%] is the corresponding weight fraction obtained by co-pyrolysis (calculated as defined above). Thus, *YD* exceeds unity if higher product yields are obtained in the PE melt, and is lower than unity in the opposite case.

### *In situ* ESR analysis

The radical behaviour in the liquid/solid phases during BW/PE co-pyrolysis was probed by *in-situ* ESR (Fig. 2, JES-X320/ES-DVT400; JEOL RESONANCE Inc.). Mixed samples (0.05 g) with BW:PE (w:w) ratios of 100:0, 60:40, 40:60, and 0:100 were used to fill a quartz tube with an inner diameter of 4.0 mm, a wall thickness of 0.5 mm, and a length of 300 mm to a height of 4 cm. Residual air in the sample tube was removed by purging with N_2_. The tube was then inserted into the ESR apparatus and heated to the desired temperature (100, 150, 200, 250, 300, and 350 °C) by heated air. A temperature sensor was located immediately below the sample tube. The air was heated by a heater controlled by a temperature controller to achieve the desired temperature, after which it was stabilised for 2 min prior to the data acquisition. The following measurement parameters were used: microwave frequency = 9052 MHz, microwave power = 1 mW, magnetic field = 322.6 ± 10 mT, modulation width = 0.2 mT, sweep time = 30 s, and time constant = 0.03 s. MnO was used as an internal standard sample and was measured with every sample.

The spin concentrations in the BW (*N*_BW, spins-T_ [spin g^−1^]) samples at each temperature were determined as follows. Coal with a known spin concentration (*N*_coal, spins_ [spin]) and MnO (marker) were analysed at 293 K to determine the double-integrated areas of the respective peaks (*A*_coal-293K_ and *A*_marker-293K_). Subsequently, BW was analysed in the presence of the same amount of MnO at 293 K, and the double-integrated areas of BW (*A*_BW-293K_) and MnO (*A*_marker-293K_) peaks were calculated. Next, each sample was analysed in the presence of the same amount of MnO at each temperature (*T* [K]) and the abovementioned areas were re-calculated (*A*_sampe-*T*_ and *A*_marker-*T*_). Since the signal intensity decreased linearly with the temperature, it was standardised by multiplying by 293/*T*. Under the utilised conditions, the intensity of the PE-derived signal was negligible at each temperature, allowing the obtained spin concentration to be standardised relative to the BW loading (*W*_BW_ [g]). Thus, the BW spin concentrations were calculated using the following equation:

4 $${N}_{\mathrm{BW},\mathrm{spins}-T}=[{N}_{\mathrm{coal},{\rm{spins}}}\times ({A}_{\mathrm{sample}-T}/{A}_{\mathrm{marker}-T})/({A}_{\mathrm{coal}-T}/{A}_{\mathrm{marker}-T})\times (293/{T}_{{\rm{sample}}})]/{W}_{{\rm{BW}}}$$

## Supplementary information

Supporting Information
