# Supplementary material for: Beech Wood Pyrolysis in Polyethylene Melt as a Means of Enhancing Levoglucosan and Methoxyphenol Production
Source: Sci Rep. 2019 Feb 13;9:1955. doi: 10.1038/s41598-018-37146-w (PMC6374462; doi:10.1038/s41598-018-37146-w)
Supplement: Supplementary file 1 — Supporting Information [file 41598_2018_37146_MOESM1_ESM.doc]

**Supporting Information**

Beech Wood Pyrolysis in Polyethylene Melt as a Means of Enhancing Levoglucosan and Methoxyphenol Production

Shogo Kumagai ^a,*^, Kohei Fujita ^a^, Yusuke Takahashi ^a^, Yumi Nakai ^b^, Tomohito Kameda ^a^, Yuko Saito ^a^, and Toshiaki Yoshioka ^a^

*^a^* Graduate School of Environmental Studies, Tohoku University, 6-6-07 Aoba, Aramaki-aza, Aoba-ku, Sendai, Miyagi 980-8579, Japan

*^b^* JEOL RESONANCE, Inc., 3-1-2 Musashino, Akishima, Tokyo 196-8558, Japan

*Corresponding author. Email: kumagai@tohoku.ac.jp

*Sample preparation and characterisation*

Commercial BW and PE (Sigma-Aldrich, Tokyo, Japan) were ground and sieved to yield particles with a size of < 250 μm. The characteristics of the BW sample are listed in Table S1. The carbon, hydrogen, nitrogen, and sulfur content were determined using Micro Corder JM10 and Yanako MT-6 elemental analysis systems (J*-*Science Lab, Kyoto, Japan). Proximate analysis was carried out under previously reported conditions using a thermogravimetric (TG) analyser (Seiko Instruments, Tokyo, Japan, TG/DTA6200).[^1^](#_ENREF_1) The oxygen content was determined from the mass balance considerations. The hemicellulose and lignin contents of the prepared BW were determined as described in the literature, and the cellulose content was determined by subtracting the moisture, extractives, ash, hemicellulose, and lignin contents.

**Table S1**. Characteristics of the utilised BW sample.[^4^](#_ENREF_4)

| *Components* [wt%] | | | | | |
| --- | --- | --- | --- | --- | --- |
| Cellulose | Hemicellulose | Lignin | Water | Extractives | Ash |
| 41.6 | 26.5 | 23.1 | 7.0 | 1.4 | 0.4 |
| *Proximate analysis*^*^ [wt%] | | | | | |
| Water | Volatiles | Fixed carbon | Ash | | |
| 7.0 | 80.2 | 12.4 | 0.4 | | |
| *Ultimate analysis* [wt%] | | | | | |
| Carbon | Hydrogen | Nitrogen | Sulfur | Oxygen^**^ | |
| 49.9 | 6.2 | - | - | 43.9 | |
| ^*^Wet basis. ^**^Calculated as mass difference (balance). | | | | | |

The ground BW was dried at 105 °C until the sample weight stabilised. The dried sample was extracted with benzene/ethanol (2:1, v/v) using a Soxhlet extractor for 6 h, and the solvents were subsequently completely removed by heating in the oven at 105 °C. Demineralisation was carried out by washing with 0.05 M HCl/methanol at room temperature for 24 h. After filtration, the treated sample was repeatedly washed with distilled water. This procedure was repeated twice, and the demineralised sample was dried at 105 °C until the sample weight stabilised. Finally, moisture-, extractives-, and ash-free BW was obtained. The pretreatment conditions were determined based on previous reports. No change was observed in the FTIR spectra (Figure S1) of the sample before and after the pretreatment. According to previous studies, these pretreatment conditions are too mild to induce hydrolysis or alcoholysis.

All other chemicals used in this work were purchased from Kanto Chemical (Tokyo, Japan), Tokyo Chemical Industry (Tokyo, Japan), and Tanuma Sanso Shoukai KK (Sendai, Japan).

**Figure S1.** FT-IR spectrum of the original, ash-free, and extractives- and ash-free samples.

**Figure S2.** TGA curves of the BW and PE samples obtained under ramp heating from ambient temperature to 650 °C at 10 °C/min.

**Figure S3.** Experimental setup used for pyrolysis experiments.

*Product analysis by gas chromatography (GC)*

Gases were identified by gas chromatography-mass spectrometry (GC-MS) using an Agilent gas chromatograph (6890N) with a 5975C mass-selective detector and a CP-PORABOND Q column. The quantification of the gases was carried out by GC using either a flame ionisation detection (GC-FID; GC: GL Science GC4000; column: CP-PORABOND Q) or thermal conductivity detection (GC-TCD; GC: GL Science GC323; column: 30–60 mesh molecular sieves) against the standard gases.

The THF solution containing tar and oil was divided in two portions. The first part was analysed by GC-MS using a Hewlett Packard system (GC: HP6890; MS: HP5975; column: InertCap 5MS/Sil) for identification, and quantified by GC-FID using a GL Science system (GC: GC390; column: InertCap 5MS/Sil), with naphthalene used as an internal standard. The abovementioned analyses allowed us to determine the compositions of the PE-derived oil and the mainly lignin-derived tar. The second part of the THF solution was subjected to oxime-trimethylsilylation (oxime-TMS),[^9^](#_ENREF_9) and the obtained oxime-TMS derivatives were identified and quantified by GC-MS and GC-FID, respectively, thus allowing us to determine the contents of wood-polysaccharide-derived products such as levoglucosan, glycol aldehyde, and hydroxyacetone. The thus-obtained detailed weight composition of the pyrolysis products is provided in Table S2. The char and PE melt were analysed by optical microscopy (VHX-2000, Keyence, Japan), and scanning electron microscopy coupled with energy-dispersive X-ray spectroscopy (SEM-EDX; S-4800, Hitachi, Japan).

**Table S2.** Weight compositions of the identified pyrolysis products obtained from the PE, BW, and BW/PE mixtures at 350 °C.

|  |  |  | BW:PE (w:w) | | | |
| --- | --- | --- | --- | --- | --- | --- |
|  |  |  | 0:100 | 40:60 | 60:40 | 100:0 |
| ***Gas/wt%***^1^ | | | ***0.4*** | ***2.2*** | ***2.7*** | ***4.1*** |
|  |  | CO | – | 0.4 | 0.6 | 1.0 |
|  |  | CO_2_ | – | 1.5 | 1.9 | 3.0 |
|  |  | CH_4_ | – | – | + | + |
|  |  | C_2_ | + | + | + | + |
|  |  | C_3_ | 0.1 | + | + | + |
|  |  | C_4_ | + | + | + | + |
|  |  | C_5_ | + | + | + | + |
|  |  | C_6_ | 0.1 | + | + | + |
|  |  | C_7_ | + | + | + | + |
|  |  | C_8_ | + | + | + | + |
| ***Tar/wt%***^1^ | | | ***-*** | ***7.1*** | ***9.8*** | ***12.7*** |
|  | *Anhydrosugars* | | – | *3.9* | *5.0* | *6.1* |
|  |  | Levoglucosan | – | 3.7 | 4.8 | 5.6 |
|  |  | Levoglucosenone | – | + | 0.1 | 0.1 |
|  |  | 1,4:3,6-dianhydro-α-D-glucopyranose | – | 0.1 | 0.1 | 0.2 |
|  |  | 1,6:3,4-dianhydro-2-O-acetyl-β-D-altropyranose | – | + | + | 0.1 |
|  | *C_2_–C_4_ fragments* | | – | *0.2* | *0.5* | *1.0* |
|  |  | Glycolaldehyde | – | 0.2 | 0.4 | 0.9 |
|  |  | Acetaldehyde | – | + | + | + |
|  |  | Acrolein | – | + | + | + |
|  |  | Hydroxyacetone | – | + | + | + |
|  |  | Propionic acid | – | + | + | + |
|  |  | 2-butenal | – | + | + | + |
|  |  | 2-butanone | – | + | + | + |
|  | *Five-membered-ring compounds* | | – | *0.3* | *0.5* | *0.8* |
|  |  | Furan | – | 0.1 | 0.1 | 0.1 |
|  |  | 2-methylfuran | – | + | + | + |
|  |  | 2,5-dimetylfuran | – | + | + | + |
|  |  | Furfural | – | 0.1 | 0.1 | 0.1 |
|  |  | 5-methylfurfural | – | + | + | 0.1 |
|  |  | 5-hydroxymethylfurfural | – | 0.1 | 0.2 | 0.3 |
|  |  | 2,5-furandicarboxyaldehyde | – | + | + | + |
|  |  | 2,5-dihydro-5-methylfuran-2-one | – | + | + | 0.1 |
|  |  | 2-hydroxy-3-methyl-2-cycropenten-1-one | – | + | + | + |
|  |  | 2-hydroxymethylenetetrahydrofuran-3-one | – | + | 0.1 | 0.1 |
|  | *Saturated alkyl side chain methoxyphenols* | | – | *0.1* | *0.1* | *0.1* |
|  |  | Guaiacol | – | + | 0.1 | 0.1 |
|  |  | 4-methylguaiacol | – | + | + | + |
|  |  | 4-ethylguaiacol | – | + | + | + |
|  |  | 4-propylguaiacol | – | + | + | + |
|  | *Unsaturated alkyl side chain methoxyphenols* | | – | *0.2* | *0.2* | *0.3* |
|  |  | 4-vinylguaiacol | – | 0.1 | 0.1 | 0.1 |
|  |  | Eugenol | – | + | + | + |
|  |  | *Trans*-isoeugenol | – | 0.1 | 0.1 | 0.1 |
|  |  | *Cis*-isoeugenol | – | + | + | + |
|  | *Carbonyl side chain methoxyphenols* | | – | *0.3* | *0.6* | *0.7* |
|  |  | Vanillin | – | 0.1 | 0.1 | 0.2 |
|  |  | Homovanillin | – | 0.1 | 0.3 | 0.3 |
|  |  | Coniferyl aldehyde | – | + | + | + |
|  |  | Acetovanillone | – | + | 0.1 | 0.1 |
|  |  | Guaiacylacetone | – | + | + | + |
|  |  | Propiovanillone | – | 0.1 | 0.1 | 0.1 |
|  |  | Dihydroconiferyl alcohol | – | + | + | + |
|  | *Phenols* | | – | *+* | *+* | *+* |
|  |  | Phenol | – | + | + | + |
|  |  | 4-methylphenol | – | + | + | + |
|  |  | 4-propenylphenol | – | + | + | + |
|  | *Others* | | – | *2.2* | *2.9* | *3.7* |
| ***Oil/wt%***^1^ | | | ***0.4*** | ***0.3*** | ***0.3*** | – |
|  |  | C_14_ | + | + | + | – |
|  |  | C_15_ | + | + | + | – |
|  |  | C_16_ | + | + | + | – |
|  |  | C_17_ | + | + | + | – |
|  |  | C_18_ | 0.1 | + | + | – |
|  |  | C_19_ | + | + | + | – |
|  |  | C_20_ | + | + | + | – |
|  |  | C_21_ | + | + | + | – |
|  |  | C_22_ | + | + | + | – |
|  |  | C_23_ | + | + | + | – |
|  |  | C_24_ | + | + | + | – |
|  |  | C_25_ | + | + | + | – |
|  |  | C_26_ | + | + | + | – |
|  |  | C_27_ | + | + | + | – |
|  |  | C_28_ | + | + | + | – |
|  |  | C_29_ | + | + | + | – |
|  |  | C_30_ | + | + | + | – |
|  |  | C_31_ | + | + | + | – |
|  |  | C_32_ | + | + | + | – |
|  |  | C_33_ | + | + | + | – |
| ***Melted PE/wt%***^2^ | | | ***89.7*** | ***-*** | ***-*** | ***-*** |
| ***Char/wt%***^2^ | | |  |  |  | ***26.5*** |
| ***Melted PE + Char/wt%***^2^ | | | ***-*** | ***65.6*** | ***51.4*** | ***-*** |
| ***Water/wt%***^3^ | | | ***-*** | ***5.3*** | ***8.7*** | ***10.8*** |
| ***Identified Total/wt%***^4^ | | | ***90.5*** | ***80.5*** | ***72.9*** | ***54.1*** |
| –: not detected; +: < 0.05 wt%; /: skipped calculation.  ^1^Determined by GC analysis.  ^2^Determined by weight measurement.  ^3^Determined by the Karl Fischer titration. ^4^Not identified products include wax and high molecular weight compounds (THF insoluble, undetectable by GC). | | | | | | |

Figure S4. ESR spectra of (a) cellulose heated at 100, 150, and 200 °C and (b) PE heated at 400 °C.

**REFERENCES**

1 Kumagai, S. *et al.* Novel Ni–Mg–Al–Ca catalyst for enhanced hydrogen production for the pyrolysis–gasification of a biomass/plastic mixture. *Journal of Analytical and Applied Pyrolysis* **113**, 15-21 (2015).

2 Haraguchi, T. *Mokuzai no kagaku*. (Buneidou, 1985).

3 Li, S., Xu, S., Liu, S., Yang, C. & Lu, Q. Fast pyrolysis of biomass in free-fall reactor for hydrogen-rich gas. *Fuel Processing Technology* **85**, 1201-1211 (2004).

4 Kumagai, S., Fujita, K., Kameda, T. & Yoshioka, T. Interactions of beech wood–polyethylene mixtures during co-pyrolysis. *Journal of Analytical and Applied Pyrolysis* **122**, 531-540 (2016).

5 Hosoya, T., Kawamoto, H. & Saka, S. Influence of inorganic matter on wood pyrolysis at gasification temperature. *Journal of Wood Science* **53**, 351-357 (2007).

6 Asmadi, M., Kawamoto, H. & Saka, S. Pyrolysis reactions of Japanese cedar and Japanese beech woods in a closed ampoule reactor. *Journal of Wood Science* **56**, 319-330 (2010).

7 Gray, M. R., Corcoran, W. H. & Gavalas, G. R. Pyrolysis of a wood-derived material. Effects of moisture and ash content. *Industrial & Engineering Chemistry Process Design and Development* **24**, 646-651 (1985).

8 Deng, W., Zhang, Q. & Wang, Y. Polyoxometalates as efficient catalysts for transformations of cellulose into platform chemicals. *Dalton Transactions* **41**, 9817-9831 (2012).

9 Hosoya, T., Kawamoto, H. & Saka, S. Oxime-trimethylsilylation method for analysis of wood pyrolysate. *Journal of Analytical and Applied Pyrolysis* **77**, 121-126 (2006).
